# Turning the Crisis Into an Opportunity: Digital Health Strategies Deployed During the COVID-19 Outbreak

**DOI:** 10.2196/19106

**Published:** 2020-05-04

**Authors:** Pol Pérez Sust, Oscar Solans, Joan Carles Fajardo, Manuel Medina Peralta, Pepi Rodenas, Jordi Gabaldà, Luis Garcia Eroles, Adrià Comella, César Velasco Muñoz, Josuè Sallent Ribes, Rosa Roma Monfa, Jordi Piera-Jimenez

**Affiliations:** 1 Servei Català de la Salut Barcelona Spain; 2 Institut Català de la Salut Barcelona Spain; 3 Centre de Telecomunicacions i Tecnologies de la Informació L'Hospitalet de Llobregat Barcelona Spain; 4 Agència de Qualitat i Avaluació Sanitàries de Catalunya Barcelona Spain; 5 Fundació TIC Salut i Social Mataró Spain; 6 Open Evidence Research Group Universitat Oberta de Catalunya Barcelona Spain

**Keywords:** digital health, eHealth, telemedicine, COVID-19, coronavirus, SARS-CoV-2, public health, policymaking

## Abstract

Digital health technologies offer significant opportunities to reshape current health care systems. From the adoption of electronic medical records to mobile health apps and other disruptive technologies, digital health solutions have promised a better quality of care at a more sustainable cost. However, the widescale adoption of these solutions is lagging behind. The most adverse scenarios often provide an opportunity to develop and test the capacity of digital health technologies to increase the efficiency of health care systems. Catalonia (Northeast Spain) is one of the most advanced regions in terms of digital health adoption across Europe. The region has a long tradition of health information exchange in the public health care sector and is currently implementing an ambitious digital health strategy. In this viewpoint, we discuss the crucial role digital health solutions play during the coronavirus disease (COVID-19) pandemic to support public health policies. We also report on the strategies currently deployed at scale during the outbreak in Catalonia.

## Introduction

Policymakers increasingly explore, accept, and apply information and communication technology (ICT) as part of health care systems. This shapes the way citizens and patients access and interact with the systems. The pathway to digital health (electronic health or eHealth) is a cultural transformation of the traditional construct of health care that encompasses multiple features, including widespread access to electronic health records, remote monitoring solutions, patient portals, wearable technologies, mobile health apps, data analytics, as well as other disruptive technologies [1].

For years, eHealth solutions have raised expectations on the cost savings associated with a reduction in travel to health care facilities and prevention of unplanned admissions due to regular check-ups [2]. In the last decade, the health care ecosystem has remarkably progressed in this direction; however, the multilevel complexity of eHealth implementation [3] is holding back the widespread use of ICT in routine practice [4].

With roughly 7.5 million inhabitants, Catalonia (Northeast Spain) has been considered a forerunner of eHealth adoption in Europe. Since 2009, a robust information exchange deployment has allowed health care providers within the public health system to share clinical information [5-7]. Currently, the region is implementing a comprehensive digital strategy—it is just one of the few ambitious initiatives that is transforming health information systems in Europe [7,8].

Worldwide, Spain is one of the most affected countries by the coronavirus disease (COVID-19) outbreak [9]. As of April 30, 2020, confirmed cases and deaths in Catalonia amounted to 54,324 and 5897, respectively. However, mathematical models predict a worsening of this scenario in the forthcoming days, which may lead to the saturation of the health care system due to the lack of intensive care specialists and complete occupation of intensive care unit (ICU) beds [10].

While clinical staff remains at the frontline to protect citizens from the pandemic, nonclinical actors like engineers, bioengineers, data scientists, and other ICT-related professionals are now taking the lead in fighting intensively to slow down the infection rate by deploying digital health solutions. In this context, the deployment of eHealth plays a major role in supporting public health policy [11,12].

**Figure 1:**
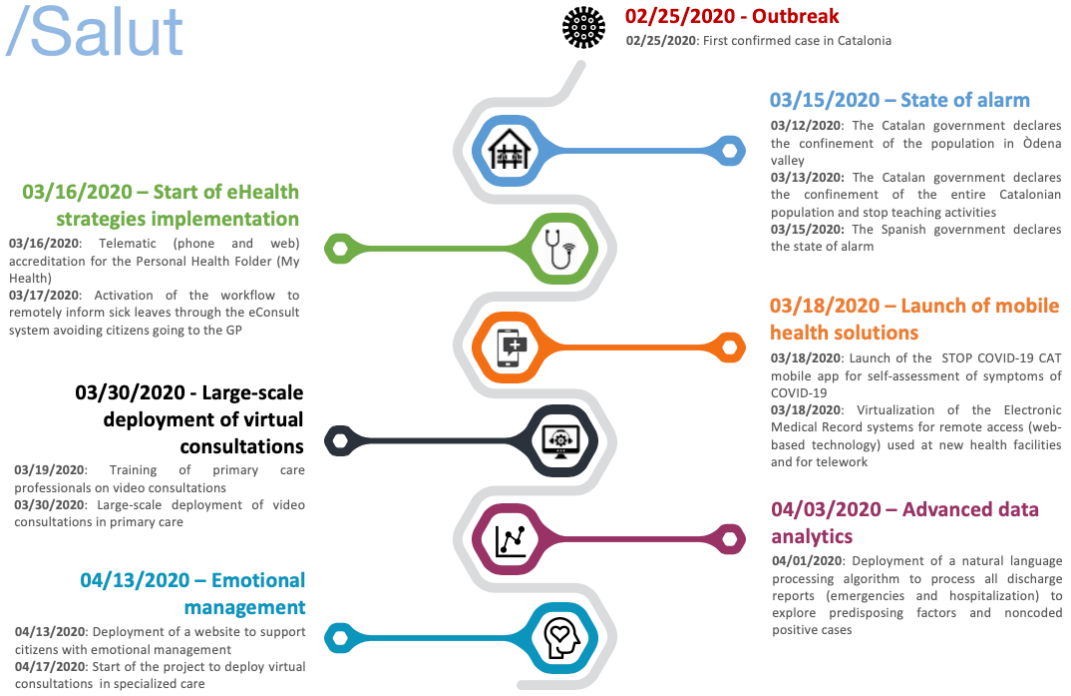
Timeline of the digital health strategies deployed in Catalonia since the onset of the coronavirus disease (COVID-19) outbreak. eHealth: electronic health; GP: general practitioner.

The objective of this viewpoint is to present the eHealth strategies adopted by the Catalonian Department of Health and the Catalan Health Service. These strategies aimed to avoid nonessential patient contact with the health care system and to improve control and diagnosis of COVID-19 (see Figure 1 for a detailed timeline). We report on the different strategies, the main objectives they are targeting, and the impact on stakeholders (Table 1).

**Table 1:** List of digital health strategies implemented during the coronavirus disease (COVID-19) outbreak in Catalonia.

Strategy	Aims and expected benefits	Impact on stakeholders
1. Facilitation of citizens’ registration on the Catalan Personal Health Folder (“My Health”) [13] by creating a specific call center and enabling a webform for self-registration	Establish a formal and secure communication channel between the citizen and the health care professional Decrease nonessential visits to health centers by citizens Reduce infection risk for both citizens and health care professionals	Citizens: burden of getting used to a new communication channel; reduction in face-to-face visits Health care providers: change of care delivery model (ie, organizational and technical workflows); training of clinical staff; change management (ie, attitudes of reluctant professionals) Policymakers: new appointment management system; cybersecurity management; guaranteeing equity on access
2. Expansion of the virtual visits system (“eConsult”) [14] by allowing the physician to appoint a videoconferencing session with the patient directly from the patient’s EMR^a^ in both primary and specialized care	Establish a synchronous and asynchronous communication channel between the citizen and the health care professional Decrease nonessential visits to health centers by citizens Reduce the infection risk for both citizens and health care professionals Avoid increases in waiting lists Ensure care continuity Avoid increase in stress in health care professionals due to not being able to attend to their patients	Citizens: burden of getting used to a new communication channel; reduction in face-to-face visits Health care providers: change of care delivery model (including organizational and change management); training of clinical staff; adaptation to new technologies (ie, integration with new platforms and acquisition of new hardware such as webcams and headphones) Policymakers: development of new technologies and design of new financing models (ie, recognition of virtual visits as a billable service)
3. Development of a mobile health app for self-assessment of the disease (STOP COVID19 CAT) [15], which includes geolocation of patients	Create a heat map of the most affected areas Stratify patients and proactively contact high-risk individuals (Emergency Services of Catalonia) Substitute for the lack of COVID-19 tests	Citizens: burden of getting used to a new technological channel Policymakers: development of new technologies; definition of new service models; facilitate the acceptance and motivation of citizens for using the mobile health app
4. Enabling of web access to EMRs throughout virtualization technologies	Ensure that health care professionals who are working in external consultations can continue their work from home (telework) during the lockdown period Ensure a smooth deployment of EMRs in emergency facilities (eg, hotels and pavilions) Avoid increases in waiting lists Ensure care continuity	Health care providers: change of care delivery model (including organizational and change management); training of clinical staff; adaptation to new technologies Policymakers: development of new technologies; deployment at scale throughout the region (including multiple organizations such as hotels and City Councils)
5. Reduction of bureaucratic barriers in health care processes by (a) allowing patients to access their sick leave forms in their personal health folder (“My Health”); (b) allowing pharmacies to access medication plans through the electronic prescription system of Catalonia in order to reduce the burden of citizens and primary care centers; (c) automatically extending chronic medication plans (eg, oral anticoagulant therapy)	Decrease nonessential visits to health centers by citizens Reduce the infection risk for both citizens and health care professionals	Citizens: burden of getting used to a new communication channel; reduction in face-to-face visits Policymakers: development of new technologies and organizational workflows within the health care ecosystem (ie, pharmacies)
6. Reporting of the day-to-day status of patients in nursing homes (private and public) through web service technology	Ensure the availability of near real-time data to make informed decisions Identify nursing homes with a high concentration of COVID-19 diagnosed patients Ensure accurate planning of actions and allocation of resources (ie, new ICU^b^ beds and isolation facilities)	Health care providers: development of new technologies (ie, integration with the National Health Service system) Policymakers: development of new technologies and organizational workflows within the health care ecosystem (ie, nursing homes)
7. Use of data analysis techniques to: (a) predict the necessary number of ICU beds to prevent overburdening the health care system (using predictive modeling techniques); (b) automatically analyze emergency and hospitalization reports to explore predisposing factors and noncoded positive cases (using natural language processing techniques)	Avoid the collapse of the health system due to a lack of hospitalization and ICU beds Ensure accurate planning of actions and allocation of resources Enable research to advance the knowledge of the disease	Policymakers: development of new technologies; incorporation of new professional roles (ie, data scientists)
8. Management of the emotional status of citizens by deploying a web portal (“Emotional Management”) [16]	Ensure a stable emotional status of the population Provide a tool for self-evaluation in order to identify risk cases and proactively contact the at-risk individuals Provide a trusted source of information resources Provide the contact information of professional (emergency) services lines	Policymakers: development of new technologies and organizational workflows within the health care ecosystem (ie, professional psychology services)

^a^EMR: electronic medical record.

^b^ICU: intensive care unit.

**Figure 2:**
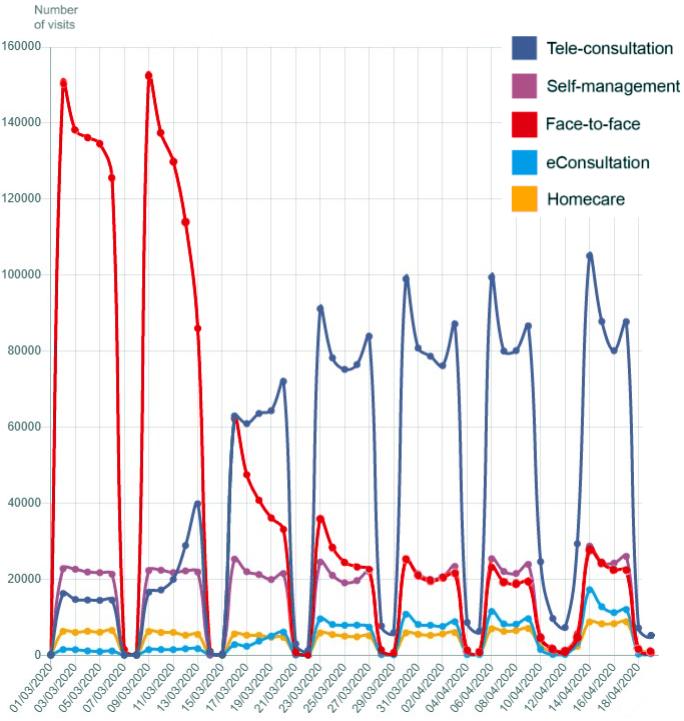
Primary care visits compared to other care delivery methods in Catalonia for the period March 01, 2020, and April 19, 2020.

Preliminary results related to the implementation of the abovementioned strategies show a strong paradigm shift from face-to-face visits to virtual consultations in primary care. Figure 2 shows how face-to-face visits have reduced drastically since the start of the Catalonian lockdown on March 16, 2020. Face-to-face visits have been systematically replaced by both tele-consultations and eConsultations (electronic consultations), which present a sustained growth over the observed period.

Adoption of digital health technologies can also be observed in the increased number of visits to and new registrations on the Catalan Personal Health Folder. Table 2 shows the development of metrics between April 2019 and April 2020 (up to April 20, 2020). In March and April 2020, the records clearly exceed the annual average.

Even though Spain and Catalonia have now passed the peak of the COVID-19 outbreak at the time of writing [17], we continue to observe an increase in the adoption of the digital health solutions deployed by the Catalonian health care system. The present context indicates a continuation of the implementation processes. In fact, the current situation is unprecedented; many adoption barriers have disappeared while at the same time health care providers and professionals are demanding more and more technologies.

The COVID-19 pandemic has prompted a sudden turning point in the adoption of eHealth strategies in Catalonia. We expect that the changes we achieved over the last few weeks will be sustained even after the pandemic is over.

**Table 2:** Number of users who accessed the Catalan Personal Health Folder and new registrations for the period April 2019 to April 2020 (up to April 20, 2020).

Date	Users who accessed the Catalan Personal Health Folder, n	New users, n
April 2019	280,001	17,026
May 2019	323,035	20,400
June 2019	293,691	15,798
July 2019	319,622	18,002
August 2019	292,248	12,475
September 2019	303,754	16,547
October 2019	376,081	21,699
November 2019	353,523	20,220
December 2019	319,021	16,022
January 2020	384,290	19,434
February 2020	390,836	21,397
March 2020	649,992	52,698
April 2020	488,207	48,862

## Lessons Learned and Next Steps

Below, we provide a list of lessons learned in the context of COVID-19 and future steps that should be taken:

1. The high pressure on the health care system in a situation of extreme crisis has been an outstanding driver of change. We analyzed the scenario to facilitate the adoption of eHealth technologies within our health system.
2. A long-term digital health strategy has proven to be the foundation for the accelerated change process. A good example of this is the unique EMR system we use in our primary health care system, which fostered the rollout of innovations faster than within a fragmented EMR ecosystem.
3. Having a very strong community and primary health care system has allowed us to implement different ICT strategies quickly by taking advantage of close interactions with the population.
4. ICT tools have been shown to be the main driver for decreasing health-related bureaucratic processes. This has allowed us to save professional staff time while avoiding nonessential visits by citizens to health centers and decreasing infection risks for both citizens and health care professionals.
5. No complaints against this comprehensive ICT deployment strategy have been received or noticed from health providers or citizens.
6. The deployment of ICT-enabled solutions should be accompanied by financial incentives for health providers in order to remove the financial barriers of adoption. Payment systems should adapt to facilitate easier ICT adoption.
7. Closer collaboration between health and social care services will be required in the future. The pandemic outbreak has shown us that coordination between both areas (ie, nursing homes and residential care) could be greatly improved by a stronger deployment of ICT (ie, access to primary care EMRs and/or deployment of telemonitoring solutions for residents).
8. We foresee many opportunities to further develop the virtual care model with more complex use case scenarios (ie, complex chronic needs). Current acceptance and need of ICT-enabled solutions has opened a window to further deploy the model in a system that has traditionally preferred face-to-face contact.
9. The ICT implementation may have avoided overcrowded health centers and, in consequence, lower infection and death rates. We need to further explore the impact of these deployments.
10. It is of outmost importance to assess how sustainable the adoption of the implemented digital health solutions on a long-term basis will be. We will continue monitoring the different implementation processes in order to assess use over time.

## Abbreviations

COVID-19: coronavirus disease
eConsultation: electronic consultation
eHealth: electronic health
EMR: electronic medical record
ICT: information and communication technology
ICU: intensive care unit
